# Genomic analysis quantifies pyroptosis in the immune microenvironment of HBV-related hepatocellular carcinoma

**DOI:** 10.3389/fimmu.2022.932303

**Published:** 2022-07-28

**Authors:** Jiarui Li, Jinghui Yu, Ting Zhang, Xingyu Pu, Yilan Li, Zhongjun Wu

**Affiliations:** ^1^Department of Hepatobiliary Surgery, The First Affiliated Hospital of Chongqing Medical University, Chongqing, China; ^2^Department of International Business, College of economics, Fudan University, Shanghai, China; ^3^Department of Phase I Clinical Trial Ward, The First Affiliated Hospital of Chongqing Medical University, Chongqing, China; ^4^Department of Liver Surgery and Liver Transplantation Center, West China Hospital of Sichuan University, Chengdu, China

**Keywords:** hepatitis B virus, hepatocellular carcinoma, pyroptosis score, fisetin, tumor immune microenvironment, anti-PD-L1 treatment

## Abstract

Pyroptosis, a way of pro-inflammatory death, plays a significant part in the tumor microenvironment (TME). A recent study has shown that the hepatitis C virus changes the TME by inducing pyroptosis against hepatocellular carcinoma (HCC). However, compared to TME in hepatitis C virus-infected HCC, the exploration of immune characteristics and response to immunotherapy associated with the pyroptosis phenotype is still insufficient in hepatitis B virus-related HCC (HBV-HCC). Our study constructed pyroptosis-score (PYS) *via* principal-component analysis (PCA) to unveil the link between pyroptosis and tumor immunity in 369 HBV-HCC patients. Compared with the low-PYS group, subjects with higher PYS were associated with poor prognosis but were more susceptible to anti-PD-L1 treatment. In addition, we found that PYS can serve independently as a prognostic factor for HBV-HCC, making it possible for us to identify specific small molecule drugs with a potential value in inhibiting tumors *via* targeting pyroptosis. Also, the target genes predicted by the Weighted gene co-expression network analysis (WGCNA) and pharmacophore model were enriched in the HIF-1 signaling pathway and NF-kB transcription factor activity, which were related to the mechanism of inflammation-driven cancer. The PYS is extremely important in predicting prognosis and responses to immunotherapy. New treatment strategies for inflammation-driven cancers may be found by targeting pyroptosis.

## Introduction

Chronic hepatitis B virus (HBV) infection is one of the leading causes of liver cancer, accounting for 50-80% of all cases globally (1). Most viruses, such as HBV DNA, RNA, and similar viral proteins, have become less dangerous due to widespread immunization and antiviral medication. However, the persistence of cccDNA makes it challenging to cure HBV with current procedures. There is a chance that some viral risk factors existed or occurred before the antiviral medication. Despite undetectable HBV DNA, viral risk factors such as HBV integration and HBV mutation may persist and contribute to the advancement of HCC.

Pyroptosis is a type of programmed cell death that relates to inflammatory cells death. Pyroptosis promotes the activation of several caspase family members, including caspase-1, through inflammasomes, causing a variety of gasdermin family members, including gasdermin-D, to shear and multimerize to generate 12-14 nm membrane holes, which is the basis of antitumor action. After cell rupture, pro-inflammatory cytokines and immunogenic chemicals are released, causing the cells to expand and eventually melt, promoting immune cell activation and infiltration (2–4). Since one of the characteristics of tumors is to avoid apoptosis, the induction of pyroptosis is of paramount significance in treating anti-apoptotic tumors. In earlier studies, Wei et al. found that the deletion of NLRP3 inflammasome is closely related to the development of hepatocellular carcinoma, while 17β-estradiol inhibits hepatocellular carcinoma *via* activating NLRP3 (5, 6). In addition, Wei et al. also found that inhibiting autophagy can accelerate the pyroptosis of hepatoma cells (7).

On the other hand, aberrant pyroptosis may have a role in fostering the establishment of the tumor immune microenvironment (TIME) due to its inflammatory character. Like many other cancers, HCC is highly heterogeneous. Although more and more immune checkpoint inhibitors have been approved to improve chemotherapy sensitivity in HCC, the outcome can still be inaccurate. Patients who have similar disease phenotypes do not mean that they have the same molecular etiology, so they are very likely to have different immunotherapy responses. In this case, stratifying patients at the molecular level will help formulate the most effective treatment options. Therefore, it is clinically significant to determine what patients benefit from immunotherapy.

Hepatitis B infection is the main cause of HCC, but it is unknown whether pyroptosis could be a potential phenotype for prognosis in HBV-HCC. Our study has constructed two subtypes of pyroptosis, closely related to different prognosis and tumor microenvironment characteristics. Unlike prior studies, we propose using the PYS to measure each HBV-HCC patient’s pyroptosis phenotype. The mRNA expression profile of pyroptosis-related genes (PRGs) is used in this technique. We are convinced that this system will help medical workers make more effective decisions in the comprehensive management of patients.

## Materials and methods

### Hepatocellular carcinoma dataset acquisition

Open access gene-expression data and complete clinical information were retrieved from Gene-Expression Omnibus (GEO; https://www.ncbi.nlm.nih.gov/gds/) and the Cancer Genome Atlas (TCGA; https://portal.gdc.cancer.gov/) database on January 6, 2022. Based on clinical data and previous literature reports, our study collected 6 eligible HBV-HCC cohorts (GSE14520, GSE141198, GSE141200, GSE10141, GSE140901, and TCGA-HBV-LIHC) with survival information for further comprehensive analysis (8–10). Fragments per kilobase million (FPKM) values of TCGA-HBV-LIHC datasets (containing 95 HBV-HCC patients) were converted into transcripts per kilobase million (TPMs). All the genes expression analysis was generated by using R with the log (2) (TPM + 1). The “ComBat” algorithm was executed when merging different datasets to reduce the likelihood of batch effects from non-biological technical biases. The complete clinical information of 369 HBV-HCC patients is shown in **Table S1**.

### Consensus clustering analysis based on PRGs

To identify unique pyroptosis clusters, we employed an unsupervised clustering algorithm to classify patients based on the expression profiles of 32 PRGs (**Table S2**) retrieved from previous reviews (11–14). The “ConsensuClusterPlus” package was performed to ensure the stability of the classification (method=“ConsensusClusterPlus”, clusterAlg=“pam”, distance=“euclidean”) (15). The subtypes, according to PRGs, were validated using principal component analysis (PCA).

### Single sample gene-set enrichment analysis

To observe the intrinsic link between different subtypes and the immune microenvironment, the degree of enrichment for 23 immune cells and 13 immunological activities in each HBV-HCC sample using the gene set variation analysis (GSVA) program (method=“gsva” and kcdf = “Gaussian”) was measured (16). A Kruskal-Wallis test was also used to assess subtypes’ immune infiltration and functions.

### Prognostic differential expression genes associated with the pyroptosis subtypes

The eligible 369 HBV-HCC patients were classified into different pyroptosis subtypes, and DEGs were identified by employing the “limma” package. We used the “clusterProfiler” package to implement Gene Ontology (GO) and Kyoto Encyclopedia of Genes and Genomes (KEGG) analysis for DEGs (17). The Cox regression analysis was then conducted to discriminate prognosis-related genes among the DEGs, in which *P*< 0.05 was considered eligible.

### Dimension reduction and calculation of PYS

Based on the expression profiles of prognostic DEGs, we classified the subjects into different gene clusters using unsupervised clustering analysis, which was verified by PCA. After that, the dimension reduction of the prognostic pyroptosis-related gene clusters was carried out by the Boruta algorithm. Principal component 1, as the gene cluster score, was obtained through the application of PCA. A formula akin to gene expression grade (18) was used to estimate PYS for each sample as follows:


$$\text{PYS}=\sum \text{PC}1_{\text{I}}-\sum \text{PC}1_{\text{II}}$$

In this formula, PC1_I_ means the first component of gene cluster I, while PC1_II_ represents the first component of gene cluster II.

### Evaluation of the PYS model

By applying the “timeROC” package, the accuracy of our model was evaluated. In contrast to the PYS model, we established nomograms that could predict patients’ 1-,3- and 5-year overall survival using the “rms” package.

### Immune landscape of PYS model

To assess the abundances of immune cell subpopulations and immune functions of the tumor microenvironment between the low- and high-PYS groups, functional enrichment was conducted *via* ssGSEA. Besides, we compared the stromal cells and immune cells scores of HBV-HCC patients by applying the “ESTIMATE” algorithm (19).

### Assessment of tumor mutation burden of PYS model

To identify the driver genes linked to TMB, we used the “maftools” program to calculate the sum of non-synonymous mutations in TCGA-HBV-LIHC data and compare somatic changes across high and low PYS groups.

### Drug and immune checkpoint blockade sensitivity

The sensitivity to chemotherapy was evaluated utilizing the Genomics of Drug Sensitivity in Cancer (GDSC; https://www.cancerrxgene.org/) database (20). Then, the half-maximal inhibitory concentration (IC50) was quantified using the “pRRophetic” package (21). We also used the Cancer Imaging Archive (TCIA; https://tcia.at/) website to forecast the therapeutic benefits of immune checkpoint blockade therapy between the low and high PYS groups (22).

### External validation of the immunotherapy based on PYS

The same protocols as the previous analysis to get the PYS of GSE140901 and the Kaplan-Meier survival curves were displayed by the “SurvMiner” package to compare the overall survival between low- and high-PYS groups after anti-PDL1 Immunotherapy of HBV-HCC patients.

### Detection of key co-expression modules applying WGCNA

We constructed the co-expression networks of the TCGA-HBV-LIHC and GSE14520 cohort, respectively, using the “WGCNA” package (minModuleSize = 50) (23). Function *pickSoftThreshold* was used to build a scale-free network where soft powers β = 2 and 3 were selected for TCGA-HBV-LIHC and GSE14520. The remaining steps are consistent with those described by Langfelder and Horvath (23).

### The excavation of compounds for small molecules based on PYS

The “limma” software was used to detect DEGs between low- and high-PYS groups for further analysis, with significant differential expression set at |log_2_FC|≥1 and FDR<0.05. Following that, using the “VennDiagram” program, the intersection of DEGs and co-expression genes retrieved from the co-expression network was used to evaluate prospective prognostic targets (24). After that, we uploaded the overlapping parts of genes into the Connectivity map (CMap; http://portals.broadinstitute.org/cmap/) website (25), and the parts of the gene expression changes caused by drugs that whether are similar or opposite to these differential gene expression profiles are identified, to screen for drugs that potentially regulate PYS. Candidate small molecular compounds whose mean is greater than 0.7 were eligible. Furthermore, we downloaded the 3D structures of compounds from PubChem (https://pubchem.ncbi.nlm.nih.gov) database (26) and obtained the molecule and pharmacophore model from PharmMapper (http://www.lilab-ecust.cn/pharmmapper/) (27). Enrichment analyses for GO and KEGG based on the specific compound’s potential target genes were performed using the “clusterProfiler” package.

### Cell culture

The human HepG2 hepatoma cell line was purchased from the Cell Bank of the Chinese Academy of Sciences (Shanghai, China). HepG2 cells were incubated in DMEM medium (VivaCell, Shanghai, China), supplemented with 10% fetal bovine serum (VivaCell, Shanghai, China). Cells were cultured in a 37°C incubator containing 5% carbon dioxide.

### CCK-8, caspase-1 activity and cytokine level assay

According to the manufacturer’s protocol, cell viability was measured with a CCK-8 assay (Absin Bioscience, Shanghai, China). Succinctly, mix with 10 μl of CCK-8 solution per 90ul complete medium, add to each well of the 96-well plate, and incubate at 37°C for 1 h. The optical density is then measured at an absorption wavelength of 450 nm. The enzymatic activity of Caspase-1 in HepG2 was detected by the Caspase 1 Activity Detection Kit (Beyotime Institute of Biotechnology, Beijing, China) according to the manufacturer’s protocol based on the ability of Caspase-1 to change acetyl-TyrVal-Ala-Asp p-nitroanilide (Ac-YVAD-pNA) into the yellow formazan product p-nitroaniline (pNA). According to the manufacturer’s instructions, cytokine levels of human IL-1β in the cell culture supernatant were determined by an ELISA kit from Neobioscience Technology (Shenzhen, China).

### Inflammasome stimulation and determination of pyroptosis

For NLRP3 inflammasome stimulation, HCC cells were stimulated by 1 μg/ml lipopolysaccharide (LPS) (Sigma-Aldrich; St. Louis, MO, USA) for 24 h to pyroptosis detection mixed with or without fisetin (MedChemExpress, New Jersey, USA). The morphology of pyroptosis was examined under light microscopy.

### Statistical analysis

**Figure 1:**
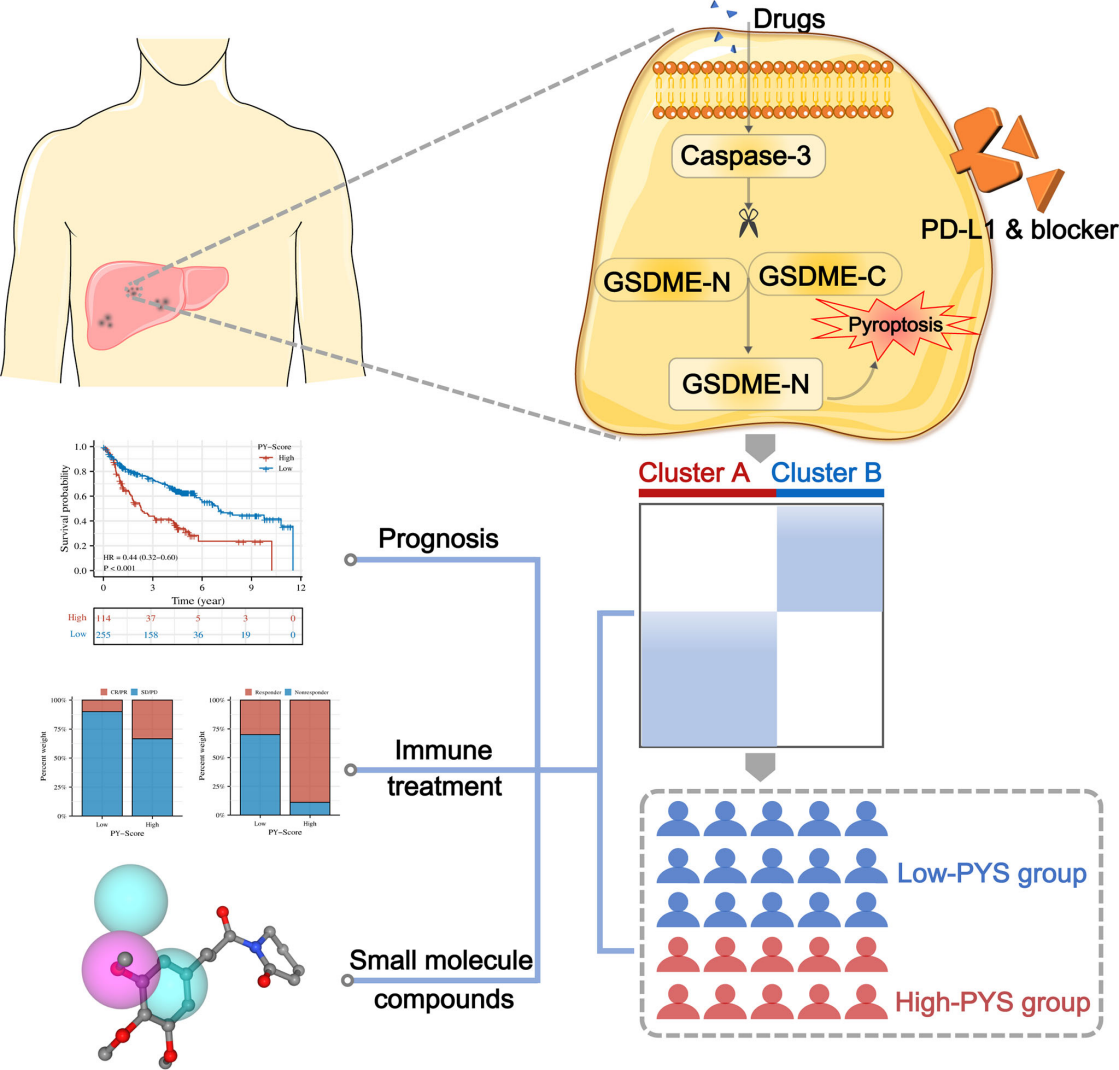
The roadmap of this study.

Statistical analysis was executed by R (version 4.0.5). The “ComBat” algorithm was conducted to diminish the batch effects. The Wilcoxon rank-sum test was used to compare two groups, and multiple comparisons were presented by the Kruskal–the Wallis test. Patients’ overall survival (OS) in different groups was compared using the Kaplan-Meier analysis and the log-rank test. The connection between OS, clinicopathological features, and pyroptosis scores was investigated using univariate and multivariate Cox regression models. The Student’s t-test was used to analyze differences between two groups, and one-way ANOVA was used when more than two groups were compared. To fix the *P*-value, we performed Bonferroni’s test. Statistical significance was defined as a two-sided *P*< 0.05. The graphic abstract of this study is shown in **Figure 1**.

## Results

### Characteristics of TIME and survival outcomes between pyroptosis phenotypes

**Figure 2:**
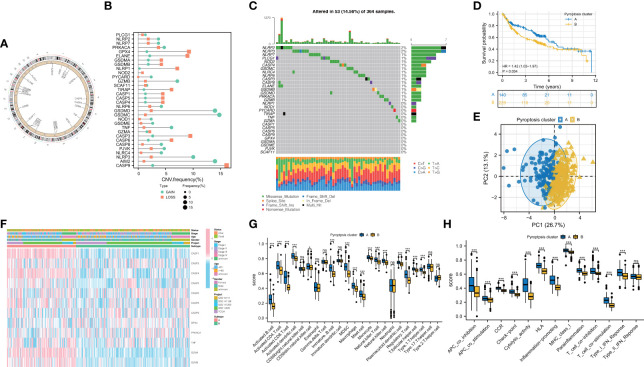
Feature and differences of pyroptosis-related molecular patterns in HBV-HCC. **(A)** The location of CNV of 23 PRGs on chromosomes. **(B)** The CNV variation frequency of PRGs and **(C)** the mutation landscape in patients from TCGA-LIHC. **(D)** Survival curves of the two subtypes. **(E)** PCA plot based on the expression of PRGs from the 369 HBV-HCC patients verified the two pyroptosis clusters, A (blue) and B (orange). **(F)** Heatmap displayed the correlation between the subtypes and clinical features. **(G, H)** The immune infiltrations **(G)** and functions **(H)** scores between the two subtypes. PRGs, pyroptosis-related genes; CNV, copy number variation. ; *P < 0.05; ***P < 0.001; ns, not statistically significant.

CNVs and TMB are not only closely linked to the occurrence and development of tumors, but also serve as an emerging biomarker for immunotherapy in diverse cancers (28–30). We found copy number variations (CNVs) in 23 of the 32 PRGs in TCGA-LIHC. The locations of CNV on chromosomes according to PRGs were shown in **Figure 2A** and mostly focused on copy number amplification (**Figure 2B**). Regarding genetic performance, 53 of the 364 samples (around 14.56%) displayed pyroptosis-related regulator mutations, among which NLRP2 had the highest frequency of mutations (**Figure 2C**). PRGs represent a set of genes that play an important role in the occurrence and development of pyroptosis. To investigate whether the degree of pyroptosis is related to the occurrence and development of HBV-HCC, we established two molecular patterns *via* unsupervised clustering analysis according to the mRNA expression profiles of 32 PRGs in 369 HBV-HCC patients (**Figures S1A–C**). As is illustrated in **Figure 2D**, the survival time of subjects in cluster A was significantly prolonged. Also, we have substantiated that these two subtypes can be discriminated by using PCA (**Figure 2E**). **Figure 2F** exhibited a heatmap of clinicopathological characteristics. Besides, we have evaluated the correlation between the subtypes and the characteristics of the tumor immune microenvironment. The results show differences in immune cell infiltration (**Figure 2G**) and immune function (**Figure 2H**) between the two subtypes. Compared with pyroptosis cluster B, activated B cells, CD8^+^ T lymphocytes, and natural killer T lymphocytes were more enriched in cluster A. Meanwhile, cytolytic activity and type I IFN response in pyroptosis cluster A have a higher score.

### Clinical and TIME features between prognostic gene clusters based on pyroptosis phenotypes

**Figure 3:**
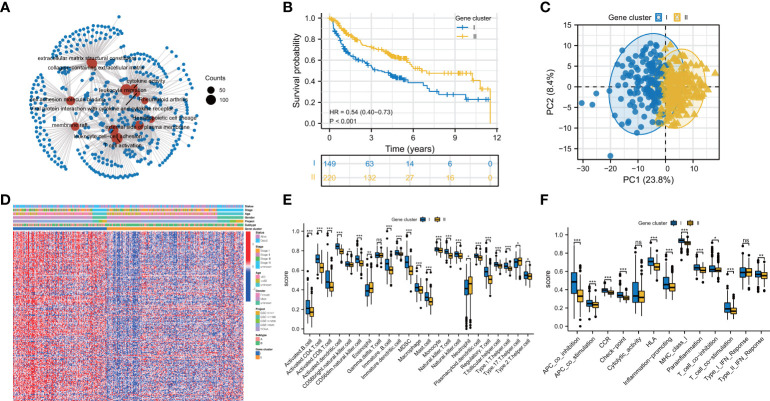
Prognosis and immune landscape between pyroptosis gene clusters for HBV-HCC patients. **(A)** GO and KEGG enrichment analysis for DEGs between two subtypes. **(B)** Survival curves of the two gene clusters. **(C)** PCA plot based on the expression of prognostic prognosis-related DEGs from pyroptosis clusters verified the two gene clusters, I (blue) and II (orange). **(D)** Clinical characteristics of gene clusters were shown by heatmaps. **(E, F)** The immune infiltrations **(E)** and functions **(F)** scores between the two gene clusters. DEGs: differentially expressed genes; *P < 0.05; **P < 0.01; ***P < 0.001; ns, not statistically significant.

To better understand the biological behaviors of these subtypes and figure out the specific score of each patient, we first performed a screen to identify the prognostic DEGs between the two subtypes. The outcomes of GO and KEGG have demonstrated that DEGs were particularly enriched in Viral protein interaction with cytokine and cytokine receptor, T cell activation, cytokine activity, and rheumatoid arthritis (**Figure 3A**). Next, we screened 229 prognostic DEGs *via* univariate Cox regression analysis (*P*<0.05, **Table S3**). Based on these prognostic DEGs, the consensus cluster analysis was performed, and thus the subjects were divided into the two gene clusters (**Figures S2A–C**). Subsequently, the prognostic significance of the gene clusters was further analyzed. As shown in **Figure 3B**, subjects within gene cluster II performed better and survived much more time.

In contrast, the ones in gene cluster I had a shorter OS time. The division of subjects based on gene clusters was validated *via* PCA (**Figure 3C**). The clinical characteristics of prognostic DEGs between the genomic clusters were visualized through a heatmap of the transcriptomic profile (**Figure 3D**). Then, we investigated whether these two gene clusters have different TIME characteristics. As shown in **Figure 3E**, the Th17 cell in gene cluster II has a higher score, while the Treg cells, Th2 cells, and dendritic cells in gene cluster I are more prominent. Intriguingly, the activated CD8^+^ T lymphocytes in gene cluster I infiltrate significantly. As tumor killer cells, CD8^+^ T lymphocytes are essential for antitumor immunity, contradicting its poor prognosis. So we looked at the expression of immune evasion-related indicators in distinct gene clusters. The results showed that T cell exhaustion-related biomarkers were up-regulated in gene cluster I, indicating that the activated CD8^+^ T cells enriched in gene cluster I and exhibited immunosuppressive effects (**Figures S3A–C**). Compared with gene cluster II, gene cluster I has higher para-inflammatory and type II interferon response scores (**Figure 3F**).

### Establishment and assessment of the pyroptosis scoring system for HBV-HCC

**Figure 4:**
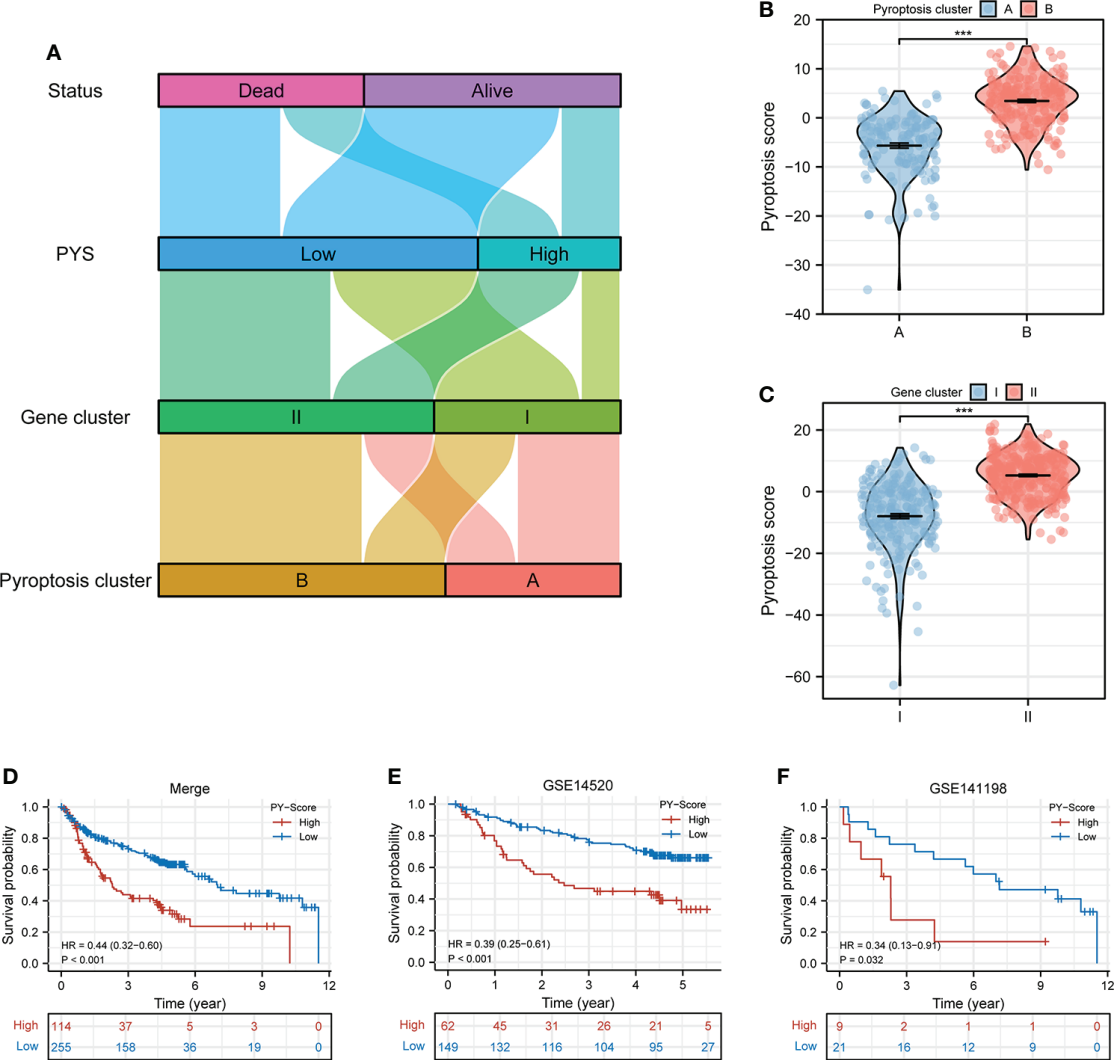
Construction of the PYS model for HBV-HCC patients. **(A)** Crosstalk between pyroptosis clusters, gene clusters, pyroptosis score, and survival status. **(B, C)** The differences in pyroptosis score in the pyroptosis clusters **(B)** and the gene clusters **(C)**. **(D–F)** Kaplan-Meier curves of patients within PYS groups in merge cohort **(D)**, GSE14520 **(E)**, and GSE141198 **(F)**. PY, pyroptosis score. ***P < 0.001

We next calculated the PYS for each subject by implementing PCA to provide useful guidance for the clinical treatment of HBV-HCC. **Figure 4A** illustrates subjects’ PYS distribution and survival status in different subtypes and gene clusters. Subjects in pyroptosis cluster B had a higher PYS (*P*<0.001; **Figure 4B**). At the same time, there was a higher PYS in gene cluster II (*P*<0.001; **Figure 4C**). As is shown in **Figures 4D–F**, subjects with higher PYS usually had a worse prognosis (*P*<0.001).

**Figure 5:**
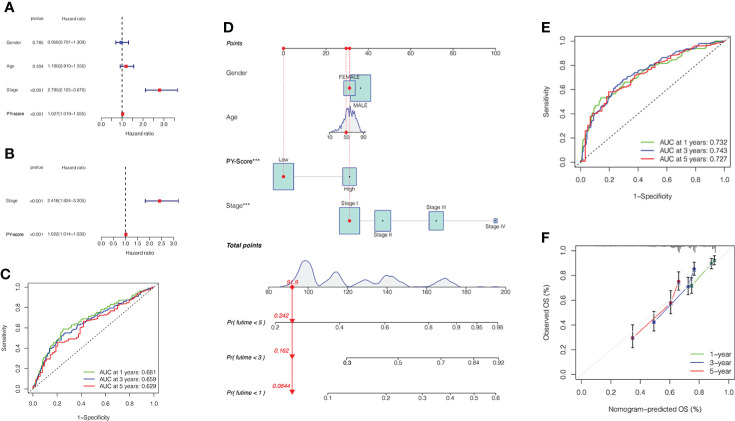
Prognostic value of PYS model for HBV-HCC patients. **(A, B)** Univariate **(A)** and multivariate **(B)** Cox regression analyses for the PYS model. **(C)** ROC curves with time dependency based on PYS. **(D)** A novel nomogram for predicting HBV-HCC prognosis based on clinical characteristics and PYS. **(E)** ROCs for the survival nomogram’s 1-, 3-, and 5-year survival time. **(F)** Calibration curve of the survival nomogram. PYS, pyroptosis score.

In an independent prognostic analysis, *via* univariate (**Figure 5A**) and multivariate (**Figure 5B**) Cox analysis, we found that PYS (HR: 1.022, 95CI: 1.014-1.030, *P*<0.001) and tumor stage (HR: 2.418, 95CI: 1.824-3.205, *P*<0.001) can serve as independent prognostic indicators of patients. **Figure 5C** showed that the predicted area under the curves (AUC) of the 1, 3, and 5-year OS are 0.681, 0.659, and 0.629, respectively, suggesting that the PYS offers an important reference value in predicting the prognosis of HBV-HCC. Meanwhile, we also constructed a survival nomogram with clinical-pathological characteristics and PYS as a “blueprint” for managing patients with HBV-HCC (**Figure 5D**). Compared with the PYS model, the survival nomogram can predict the HBV-HCC patients’ prognosis with higher validity and reliability (**Figure 5E**, **F**).

### The intrinsic relationship between the PYS and TMB

**Figure 6:**
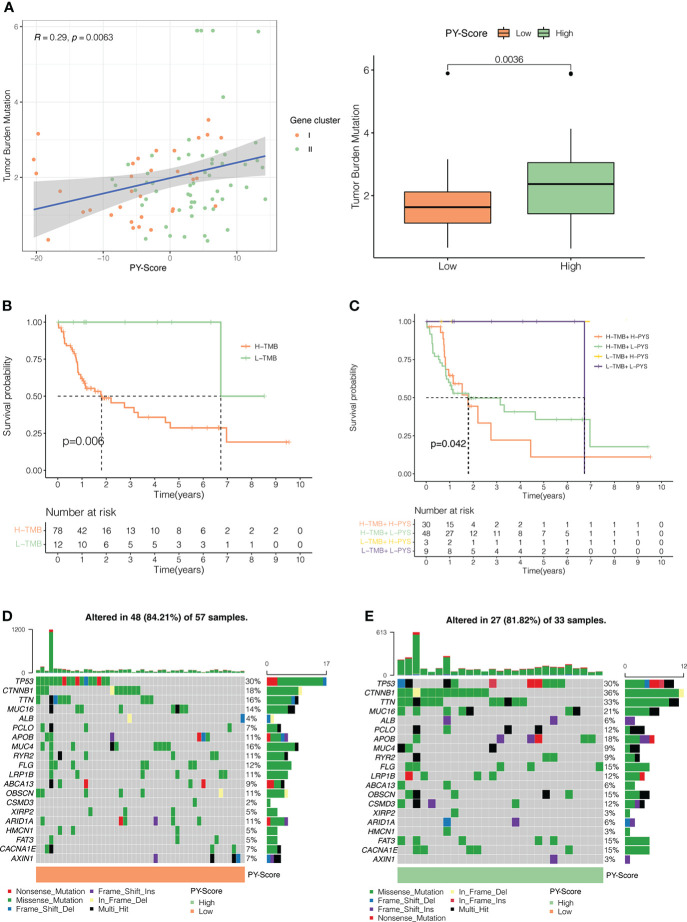
The Inner link between the PYS and TMB. **(A)** The correlation between the PYS and TMB. *P<*0.01. **(B)** Survival curves for high and low TMB groups in the TCGA-HBV-LIHC cohort. *P* =0.006. **(C)** Survival curves for HBV-HCC patients classified by TMB and PYS. The oncoplot was constructed *via* the low-PYS group **(D)** and high-PYS group **(E)**.

Many studies have concluded that TMB is not only closely related to the occurrence and development of tumors but also can predict the efficacy of immunotherapy. For example, tumors with higher mutational burdens tend to be accompanied by higher CD8^+^ T cell infiltration and improve response to PD-1 blockades, which means that TMB may determine how patients respond to immunotherapy. However, TMB alone is insufficient to predict the prognosis of patients. Therefore, we investigated the internal relationship between TMB and PYS. First, we analyzed and compared the situation of TMB in the high-PYS and low-PYS group. **Figure 6A** suggested that there was a significant correlation between TMB and PYS. Then we discovered that HBV-HCC patients with a high TMB have a worse prognosis (**Figure 6B**). To determine whether PYS can act as a predictor independent of TMB, we evaluated the synergy of PYS and TMB in the prognostic stratification of HBV-HCC. As shown in **Figure 6C**, PYS differs significantly in the high-TMB and low-TMB groups (*P*=0.042). The results indicate that PYS may serve as a biological indicator independent of TMB for predicting the efficacy of immunotherapy in HBV-HCC patients.

Subsequently, we researched the driver genes of somatic mutation in HBV-HCC, and the top 20 genes with the highest mutation frequency in the low and high PYS groups were shown in **Figures 6D**, **E**, respectively. These gene mutations prompt new ideas for the immunotherapy of HBV-HCC patients in different PYS groups.

### PYS is related to TIME features and immune checkpoint blockade therapy of HBV-HCC

**Figure 7:**
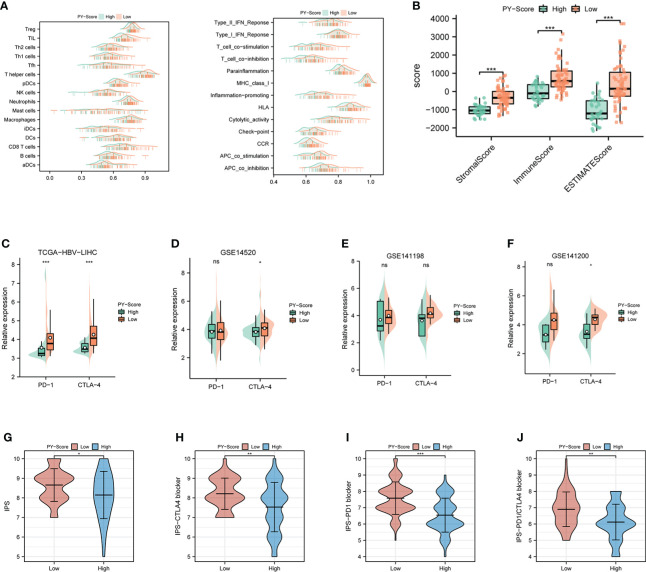
Immune landscape and immune checkpoint blockers sensitivity based on PYS. **(A)** Ridge plot of TIME-based on PYS. **(B)** tumor microenvironment scores between high- and low-PYS groups. **(C–E)** The relative expression of PD-1 and CTLA-4 between low- and high-PYS groups in TCGA-HBV-LIHC **(C)**, GSE14520 **(D)**, GSE141198 **(E)**, and GSE141200 **(F)** cohort. **(G–J)** The IPS of baseline **(G)**, CTLA-4 blocker **(H)**, PD-1 blocker **(I)**, and PD1/CTLA4 blocker **(J)**. TIME, tumor immune microenvironment; IPS,immunophenoscore; PYS, pyroptosis score. *P < 0.05; **P < 0.01; ***P < 0.001; ns, not statistically significant.

The role of pyroptosis seems to be a double-edged sword in cancer. In addition to rapidly leading to tumor regression, it can promote the development of the TIME. For further exploration of the relevance between the PYS and TIME features, the ridge plot of TIME based on PYS was shown in **Figure 7A**. Meanwhile, the immune (*P*<0.001) and stromal score (*P*<0.001) scores of patients from the low-PYS group outnumbered those of the other group (**Figure 7B**). Cellular characterization of immune infiltration suggests that tumor genotype determines immunophenotype and tumor escape mechanisms. We further worked on the expression of immune checkpoints in the high- and low-PYS groups. We found in the low-PYS group that the expression of immune escape-related biomarkers such as PD-1 and CTLA-4 were significantly up-regulated in TCGA-HBV-LIHC, GSE14520, GSE141198, and GSE141200 cohort (**Figures 7C**–**F**). The immunophenoscore (IPS) based on the expression of important components of tumor immunity between low- and high-PYS groups was assessed using the TCIA database (**Figure 7G**). Herein, we found that whether the use of CTLA-4 blocker alone (**Figure 7H**), PD-1 blocker alone (**Figure 7I**), or PD-1 combined with CTLA-4 blockers (**Figure 7J**), IPS was higher in the low-PYS group.

### Sensitivity of chemotherapy and benefits of anti-PD-L1 immunotherapy in HBV-HCC patients based on the PYS model

**Figure 8:**
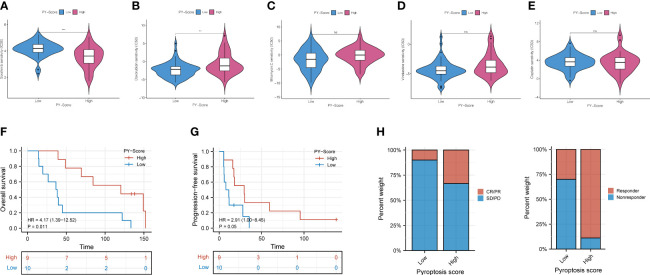
The role of PYS in assessing chemotherapy and the benefit of anti-PD-L1 immunotherapy. **(A–E)** HBV-HCC patients in the high-PYS group were associated with a lower IC50 for chemotherapy, such as Sorafenib **(A)**, while a higher IC 50 was associated with Doxorubicin **(B)**. Mitomycin C **(C)**, Vinblastine **(D)**, and Cisplatin **(E)** did not show a meaningful difference. **(F, G)** Overall survival **(F)** and progression-free survival **(G)** curves of patients within high- and low-PYS groups in the GSE140901 cohort. **(H)** Clinical response to anti-PD-L1 immunotherapy between PYS groups. CR, complete response; PR, partial response; SD, stable disease; PD, progressive disease; PFS, progression-free survival; PYS, pyroptosis score. **P < 0.01; ***P < 0.001; ns, not statistically significant.

Studies have shown that chemotherapy and some non-chemotherapeutic drugs can trigger pyroptosis in most cancers (31–33). Compared with monotherapy, combining chemotherapy and other methods is way more effective in promoting the pyroptosis of cancer cells, stimulating a stronger immune response to prevent the worsening of tumors. We then compared and analyzed the IC50 levels among 5 chemotherapy drugs, Sorafenib, Doxorubicin, Mitomycin C, Vinblastine, and Cisplatin (**Figures 8A**–**E**). Our data showed that the high-PYS group possessed a lower IC50 level of sorafenib (*P*<0.001) than the other group, while the IC50 of Doxorubicin (*P*<0.01) was exactly the opposite, whereas the high-PYS group had a higher level. A recent study has shown that sorafenib changes the tumor immune microenvironment by inducing pyroptosis against hepatocellular carcinoma. Our results further evaluated the utility of the PYS in predicting the benefit of anti-PD-L1 immunotherapy in HBV-HCC patients. First, HBV-HCC patients in the GSE140901 cohort (n=19) who received anti-PD-L1 immunotherapy were assigned to low- or high-PYS groups. It is noteworthy that high-PYS subjects performed better and lived longer than the low-PYS ones for overall survival (*P*=0.011, **Figure 8F**) and progression-free survival (*P*=0.05, **Figure 8G**). Besides, high-PYS subjects showed a higher objective response rate of anti-PD-L1 treatment than the other group in the GSE140901 cohort (**Figures 8H**). Collectively, these data indicated that anti-PD-L1 immunotherapy was likely to provide better efficacy for high-PYS subjects.

### Prospective small molecule compounds based on PYS

**Figure 9:**
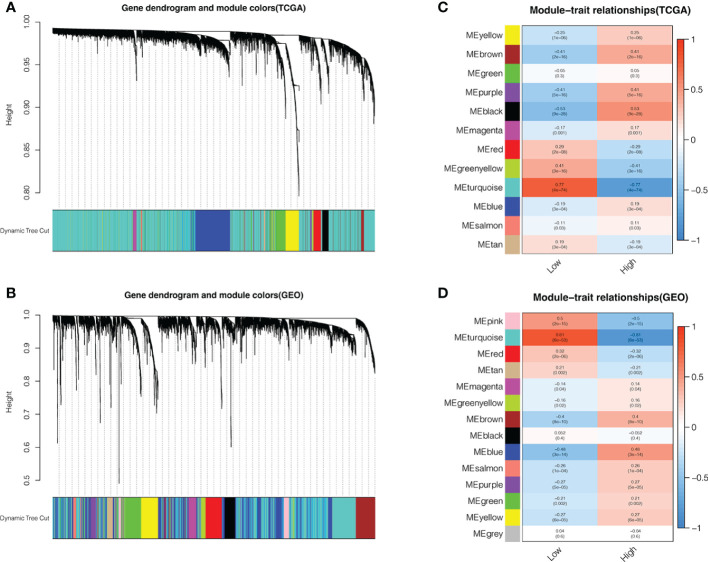
Identifying modules associated with the PYS. **(A–D)** Co-expression network module cluster dendrogram in TCGA-HBV-LIHC **(A)** and GSE14520 **(B)** cohorts. Module-trait relationships in TCGA-HBV-LIHC **(C)** and GSE14520 **(D)** cohort, respectively.

**Figure 10:**
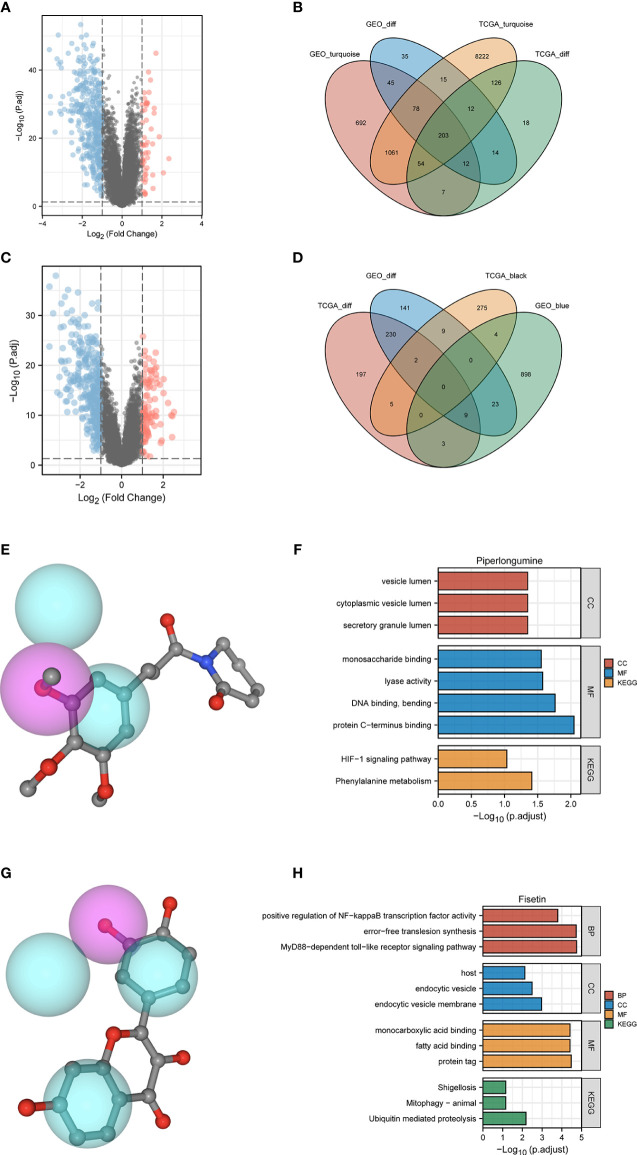
Potential small molecule compounds and function prediction. **(A–D)**. Differentially expressed genes were identified between low- and high-PYS groups in TCGA-HBV-LIHC **(A)** and GSE14520 **(B)** cohort, respectively. The Venn diagram for positive correlation **(C)** or negative correlation **(D)** with the low-PYS group. **(E)** The 3D structure diagrams of the pharmacophore for piperlongumine. **(F)** GO and KEGG for target genes of piperlongumine **(G)** The 3D structure diagrams of the pharmacophore for fiestin. **(H)** GO and KEGG for target genes of fiestin. DEGs: differently expressed genes.

We further analyzed the two groups to screen potential small molecule drugs. WGCNA package was utilized to establish a gene co-expression network from the TCGA-HBV-LIHC and GSE14520 cohorts. With colors assigned to each module, this study identified 12 modules in TCGA-HBV-LIHC (**Figure 9A**) and 14 modules in GSE14520 (**Figure 9B**). Then, we created a module-feature relationship heatmap to examine the correlation between each module and PYS, (**Figures 9C**, **D**). The turquoise module in both the TCGA-HBV-LIHC (r = 0.77, *P*= 4e−74) and GSE14520 (r = 0.81, *P* = 6e−53) were found to have the highest positive association with low PYS. On the contrary, the black module in the TCGA-HBV-LIHC (r = -0.53, *P*= 9e−28) and the brown module in GSE14520 (r = -0.4, *P*= 8e−10) were found to have the highest negative association with low PYS. According to the cut-off standard of |log_2_FC| ≥ 1.0 and adj. *P*< 0.05, 446 DEGs in the TCGA-HBV-LIHC cohort (**Figure 10A**) and 414 DEGs in the GSE14520 cohort (**Figure 10B**) were dysregulated between high- and low-PYS groups. Overlapping genes expressing was up-regulated (**Figure 10C**) and down-regulated (**Figure 10D**) in the low-PYS group were extracted for subsequent analysis. Then, we uploaded these DEGs into the CMap database. We also worked on analyzing of the underlying drug mechanisms and identified 13 potential small molecule drugs whose mean is greater than 0.7 (**Table S4**). The 3D structure diagrams of the pharmacophore for piperlongumine (**Figure 10E**) and fisetin (**Figure 10G**) were shown, respectively. After GO/KEGG enrichment analysis of target genes of piperlongumine (**Figure 10F**) and fisetin (**Figure 10H**), we observed that piperlongumine is related to lyase activity, HIF-1 signaling pathway, and phenylalanine metabolism, while fisetin is enriched in ubiquitin-mediated proteolysis, NF-kappaB transcription factor activity and MyD88 dependent TLR signaling pathway.

### Fisetin alleviated NLRP3 inflammasome activation inhibits HCC cell death

**Figure 11:**
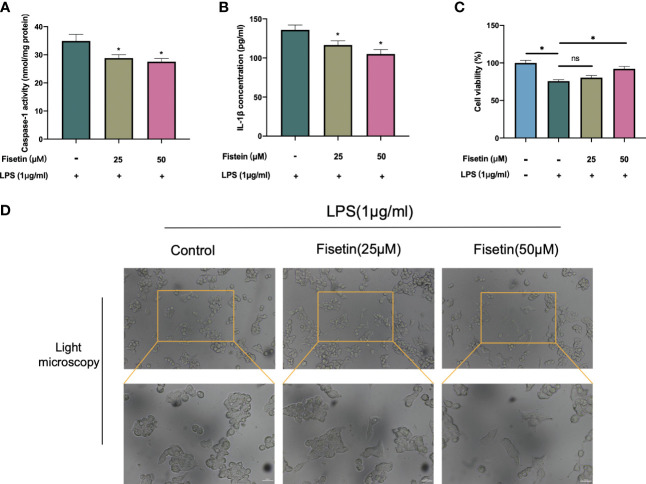
Fisetin inhibited NLRP3 inflammasome activation and alleviated HCC cell death. **(A–C)** HepG2 cells were treated with LPS(1 μg/ml) with or without fisetin at concentrations of 25 μM and 50 μM for 24 h, then the activity of caspase 1 **(A)** and IL-1β level in supernatant of culture medium **(B)** were detected by ELISA, while cell viability was detected by CCK-8 assay **(C)**. **(D)** The morphology of the HepG2 was observed with a phase-contrast microscope. *P< 0.05 by ANOVA versus control. ns, not statistically significant; Scale bars: 50 μm.

Caspase 1 activity, and IL-1β expression, were examined in this study to confirm our predicted results. In the LPS-induced pyroptosis model, the activity of Caspase 1 and the secretion of IL-1β were significantly downregulated in a dose-dependent manner in fisetin-treated HCC cells, suggesting that fisetin inhibits the activation of the NLRP3 inflammasome (**Figure 11A, B**). Besides, cell viability was detected using CCK-8 assay, as is shown in **Figure 11C**. The viability of HepG2 was gradually increased under the treatment of fisetin in a dose-dependent manner. Furthermore, we observed the morphology of the HepG2 by a light microscope. Under the induction of LPS, the cells swelled and enlarged with many bubble-like protrusions, and fisetin could alleviate the occurrence of pyroptosis to a certain extent. (**Figure 11D**).

## Discussion

In the past few years, the multi-omics analysis has finally progressed in molecular classification, which is highly related to the characteristics of genes, metabolism, immunity, and chromosome. However, the real motive for setting up a classification system is for clinical application. Therefore, stratifying patients at the molecular level will help formulate a better treatment plan and determine how effective the immunotherapy is when faced with a certain patient. Our study comprehensively analyzed the clinical value of the pyroptosis pattern in HBV-HCC. At the same time, we have compared and contrasted the prognosis, tumor immune microenvironment, and immunotherapy of patients with HBV-HCC *via* the PYS system. As a “blueprint” for managing HBV-HCC, the PYS system will provide patients with a more robust and comprehensive plan for targeted therapy.

First, we observed the CNV and TMB of PRGs in the TCGA-HBV-LIHC cohort. Unlike the staging of HBV-HCC, the classification system mainly depends on a molecular level. Certain subtypes can be targeted, and a proven method will develop specific treatment plans for liver cancer (34). Next, we derived two molecular subtypes from the mRNA expression profile of PRGs for HBV-HCC patients. The results show that subjects in pyroptosis cluster A have a better prognosis, consistent with their active immune function. To identify the prognosis-related DEGs, we then analyzed the mRNA expression profiles of different subtypes of pyrolysis and established a classification system of HBV-HCC based on genetic features. In pursuit of higher clinical value and potential application, we developed the PYS model based on these two clusters to quantify the prognostic risk and response to chemotherapy and immunotherapy, thereby providing strong evidence for clinical treatment. Above all, the PYS acted independently as a prognostic factor among clinical characteristics of HBV-HCC. The ROC curve shows its high efficiency in predicting survival rates, which provides strong support for predicting clinical outcomes.

Pyroptosis can create a milieu conducive to tumor growth as a form of pro-inflammatory death. Exploring the characteristics of the TIME between different PYS groups and improving clinical management through personalized prescription is of practical importance, according to the theory of inflammation-cancer transformation and chronic inflammation-induced carcinogenesis. As is shown in the results, patients with higher PYS had more restricted immune infiltration, especially the tumor killer CD8^+^ T cells, which was consistent with their poor prognosis. A recent study has shown that sorafenib changes the TIME by inducing pyroptosis against HCC (35). We found that patients with high- PYS were more susceptible to sorafenib. As we know, multidrug resistance is the main obstacle to cancer treatment. Thus, combined with PD-L1 blockers, sorafenib may be a potentially effective treatment for the high-PYS group. In addition, for the first time, we verified that PD-L1 blockers could improve the prognosis and clinical benefit of the high-PYS group by using an external data set.

The occurrence of pyroptosis *in vivo* indicates the prospective value of pyroptosis in regulating tumorigenesis and inducing pyroptosis has been designed to eliminate tumors. Small molecule drugs like BMS-8 and BMS-202 are crucial in tumor immunotherapy by inhibiting PD-1 and PD-L1 interaction (36, 37). Recently, Yuan et al. (38) found that Cucurbitacin B inhibits non-small cell lung cancer *via* TLR4/NLRP3/GSDMD signaling pathway. Besides, Dihydroartemisinin inducing pyroptosis *via* AIM2/caspase-3/DFNA5 axis was also reported in breast cancer (39). The above studies have shown that small-molecule drugs can potentially induce pyroptosis and regulate the TIME. Using the WGCNA and limma packages, we have obtained modules with significant differences and identified DEGs significantly related to PYS. To improve the prognosis of HBV-HCC patients, we screened 13 small molecule compounds using the CMap database. Piperlongumine can reduce colitis-associated colorectal cancer (40) and relieve sepsis by attenuating the activation of inflammasomes (41). From the enrichment results, piperlongumine acts on the HIF-1 signaling pathway and lyase activity, while fisetin regulates NF-kB transcription factor activity. The crosstalk between oncogene and tumor suppressor transcription factors such as NF-kB, STAT3, HIF-1α, and NRF2, is the mechanism of inflammation-driven cancer (42). Fisetin has been suggested to have a protective role in attenuating inflammation and HCC development for a few decades. Our previous study unveiled that fisetin relieves hepatic ischemia-reperfusion injury by inhibiting the NLRP3 inflammasome activation (43), which indicates its potential value in regulating pyroptosis. In our present experiments, the inhibition of pyroptosis by fistin was verified. Here we have demonstrated that fisetin can inhibit the activity of caspase 1 and release IL-1β, which indicates the occurrence of pyroptosis. Overall, the above studies have shown that small molecule compounds affect the occurrence and development of tumors *via* pyroptosis and inflammatory signaling pathways, which brings new options to clinical treatment.

Pyroptosis is a two-edged sword that can promote and inhibit cancer depending on the situation (14). Although we conducted our research from different angles and used several databases to confirm our conclusions, there are still some limitations. Due to the heterogeneity of HCC, targeted improvements of the PYS model may be needed for different tumor subtypes. At the same time, more data on immunotherapy related to HBV-HCC need to be established. Furthermore, the changes in the tumor immune milieu associated with the PYS model may necessitate additional explanations, such as single-cell sequencing. The small molecule compounds we screened should be tested further to understand better their link with pyroptosis, which is also the path we must strive for in the future.

## Data availability statement

The original contributions presented in the study are included in the article/**Supplementary Material**. Further inquiries can be directed to the corresponding author.

## Author contributions

ZW conceived and designed the study. JL contributed to research design, data analysis, and writing. JY revised the manuscript. TZ, XP, and YL supervise the study. All authors have read and approved the submitted version.

## Funding

This study was supported by the National Natural Science Foundation of China (No. 82170666 and 81873592), and Chongqing Technology Innovation and Application Development Special Project, Key Project, cstc2021jscx-gksbX0060.

## Conflict of interest

The authors declare that the research was conducted in the absence of any commercial or financial relationships that could be construed as a potential conflict of interest.

The reviewer SZ declared a shared parent affiliation with the author JY to the handling editor at the time of review.

## Publisher’s note

All claims expressed in this article are solely those of the authors and do not necessarily represent those of their affiliated organizations, or those of the publisher, the editors and the reviewers. Any product that may be evaluated in this article, or claim that may be made by its manufacturer, is not guaranteed or endorsed by the publisher.
