# Supplementary figures and images for: Genomic analysis quantifies pyroptosis in the immune microenvironment of HBV-related hepatocellular carcinoma
Source: Front Immunol. 2022 Jul 28;13:932303. doi: 10.3389/fimmu.2022.932303 (PMC9365940; doi:10.3389/fimmu.2022.932303)

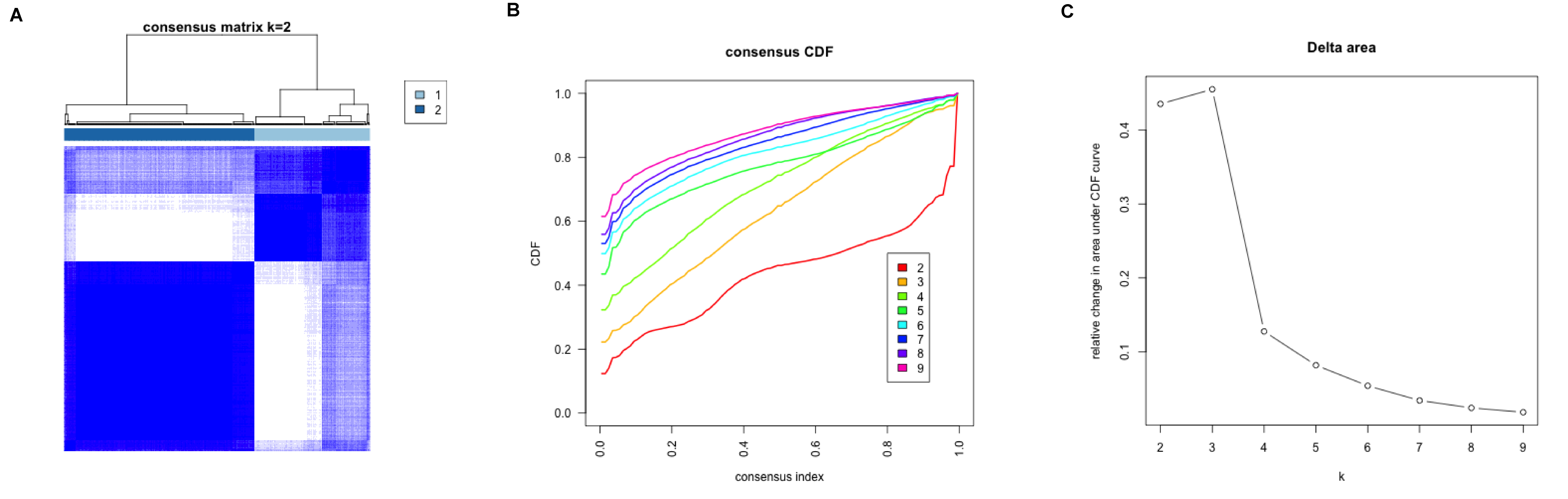

Supplement: Supplementary file 1 [file Image_1.tif]

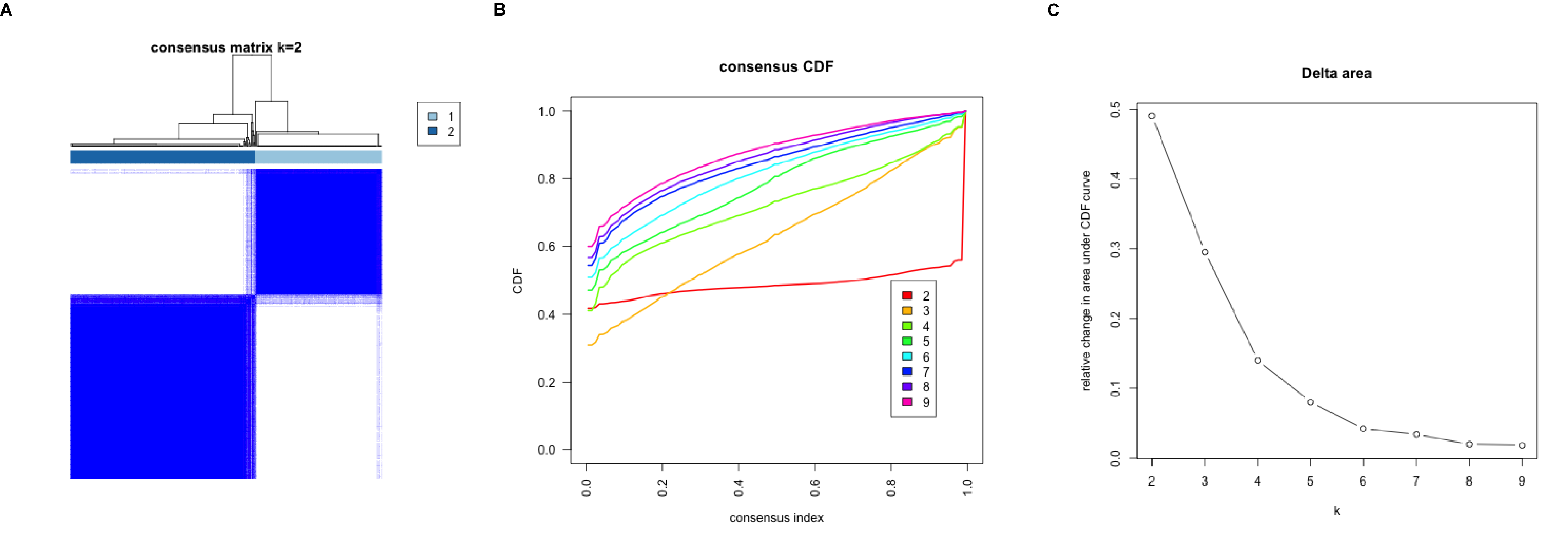

Supplement: Supplementary file 2 [file Image_2.tif]

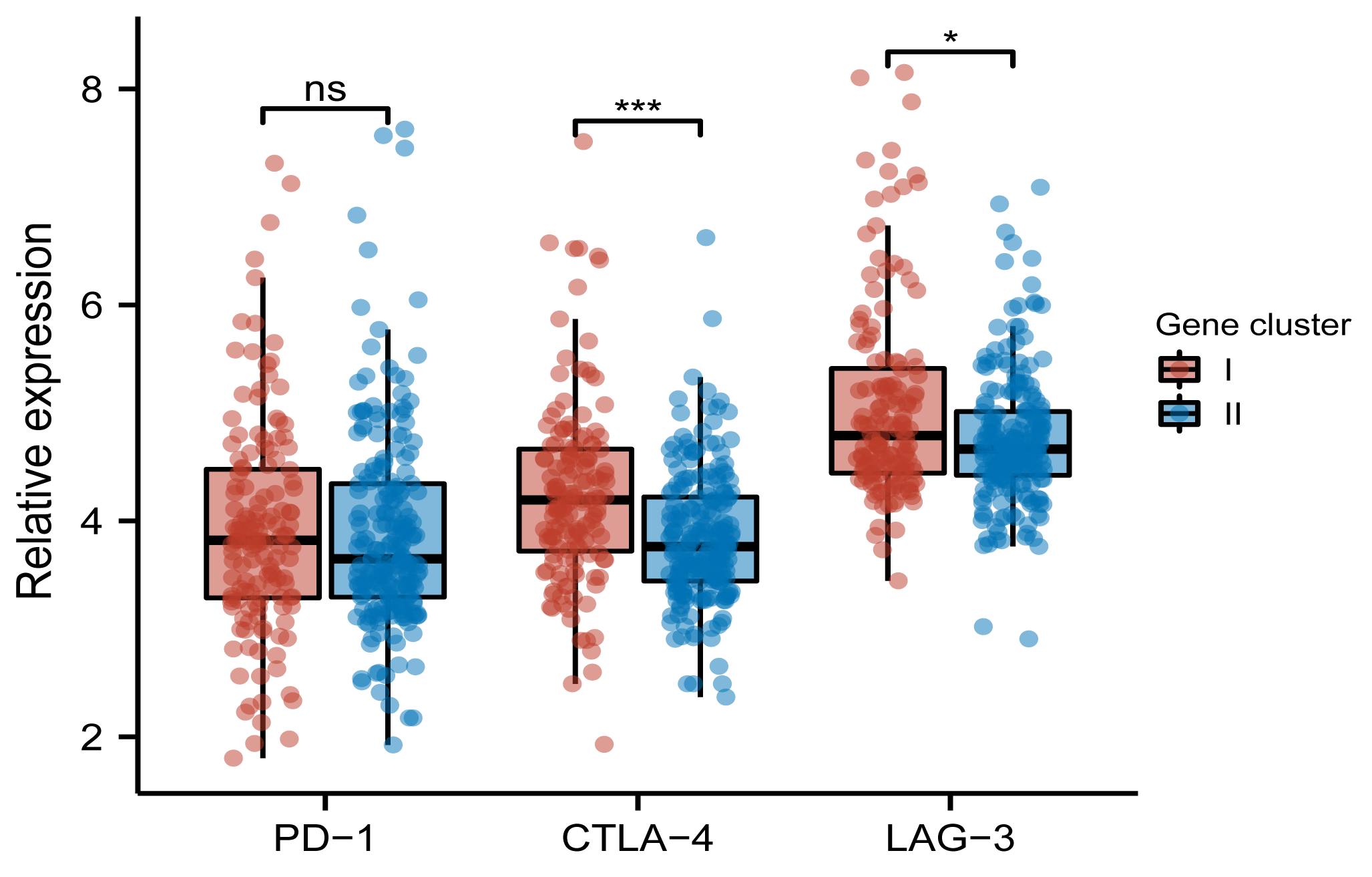

Supplement: Supplementary file 3 [file Image_3.tif]
